# Marker-assisted breeding to develop the drought-tolerant version of Sabitri, a popular variety from Nepal

**DOI:** 10.1007/s10681-017-1976-3

**Published:** 2017-07-24

**Authors:** Shalabh Dixit, Ram Baran Yadaw, Krishna Kumar Mishra, Arvind Kumar

**Affiliations:** 1 International Rice Research Institute, DAPO Box 7777, Metro Manila, Philippines; 2 Nepal Agricultural Research Council, National Rice Research Project, Hardinath, Nepal

**Keywords:** Rice, Drought, Yield, QTL, Marker-assisted backcross breeding

## Abstract

Sabitri is a rice variety grown in a large part of the rainfed areas of Nepal. It was originally developed for irrigated condition; hence, this variety suffers high yield decline under drought. Two QTLs, *qDTY_3.2_* and *qDTY_12.1_*, with large effects on grain yield under drought were identified in the Sabitri background in separate QTL mapping studies. The present study reports the development of Sabitri near isogenic lines (NILs) with combinations of these two QTLs and their characterization under drought. To do so, marker-assisted backcross breeding (MABB) was combined with phenotypic selection to develop high-yielding drought-tolerant NILs with Sabitri grain type. Apart from this, drought-tolerant variants for grain type with high yield under non-stress were identified among the developed NILs. Early days to flowering of up to 13 days and reduction in plant height of up to 13 cm as compared to Sabitri were observed in the developed NILs. Some of these NILs showed higher yield compared to Sabitri and relatively higher tolerance to drought, indicating the capture of positive alleles and interactions during the course of selection. The developed NILs possessed high yield potential which make them suitable materials for the testing of water-saving technologies in irrigated areas. Based on their performance, these NILs can be deployed in rainfed areas in Nepal and other countries of South Asia to increase yield stability.

## Introduction

Nepal is a major rice growing country in South Asia where rice was grown in 1.5 million hectares, producing 4.7 million tons in 2016 alone (http://ricestat.irri.org:8080/wrs). However, the average rice productivity in the country for the past 20 years (1996–2016) was 2.7 t ha^–1^, much lower than the world rice productivity for the same period which was 4.1 t ha^–1^(http://ricestat.irri.org:8080/wrs). To attain self-sufficiency in rice in Nepal, rice productivity needs to be increased. A clearer understanding of the rice-growing ecosystems of Nepal as well as the deployment of varieties with traits suitable to these ecosystems is also necessary to achieve the aim of rice self-sufficiency. Rice-growing ecosystems of Nepal were classified in the 2013 strategic assessment at IRRI (unpublished data). Total rice area at the time of the study was divided mainly into three different rice-growing ecosystems: irrigated (48.8%), rainfed lowland (42.6%) and rainfed upland (8.6%). Of these ecosystems, rainfed upland, dry irrigated, and shallow and intermediate rainfed areas are prone to drought of varying intensities. In addition, part of the irrigated areas dependent on water from ponds and water tanks are exposed to drought in years with less rainfall. Rainfed rice ecosystems, however, are the most unpredictable, with drought as the predominant abiotic stress that leads to a high reduction in yield. Even in a year with normal total rainfall, drought can still affect the rice crop in these areas due to the uneven distribution of rainfall during the season. Apart from this, the availability of irrigation water has been declining due to its increasing demands from the industrial and municipal sectors (Molden et al. 2007). For rice cultivation to be sustained, an increased use of water-saving technologies in irrigated areas is vital (Kumar and Ladha 2011). These scenarios highlight the need for the development of high-yielding, good quality rice varieties with drought tolerance in order to maintain yield stability under varying environmental conditions and cultivation practices. Ironically, a large proportion of rainfed areas in Nepal are still planted with drought-susceptible varieties which are developed for irrigated ecosystems. An example of such variety is Sabitri which is popular in Nepal because of its high yield and grain quality. It is grown in a large area across rainfed rice ecosystems despite its high declines in yield in cases of drought. The development of high-yielding drought-tolerant versions of this variety that carry a similar grain quality may, therefore, provide an alternative to farmers in enhancing yield stability in rainfed areas of Nepal and in other countries of South Asia that have a need for similar varieties.

Notable progress has been made in the area of genetics underlying grain yield (GY) under drought in the past decade. The establishment of grain yield as a selection criterion (Venuprasad et al. 2007, 2008; Kumar et al. 2009, 2013, 2014; Dixit et al. 2014a), the identification of large-effect quantitative trait loci (QTLs) (Bernier et al. 2007; Venuprasad et al. 2009; Vikram et al. 2011; Ghimire et al. 2012; Swamy et al. 2013; Palanog et al. 2014; Sandhu et al. 2014; Dixit et al. 2014b, c), and understanding the molecular mechanism behind different QTLs through fine mapping and genetic studies (Dixit et al. 2012a, b, 2015) have provided new tools in developing high-yielding drought-tolerant varieties. Majority of these QTLs have explained a large proportion of the phenotypic variances for GY under drought and have shown consistency of effect. Two such large effect QTLs, *qDTY_3.2_* and *qDTY_12.1_*, were reported in the Sabitri background (Yadaw et al. 2013; Mishra et al. 2013), explaining up to 23.4 and 38.8% of the total phenotypic variance, respectively. *qDTY_12.1_* was reported earlier in Vandana background (Bernier et al. 2007). Similarly, *qDTY_3.2_* was later reported in another study (Dixit et al. 2014c) confirming their robustness and effect across genetic backgrounds. *qDTY_12.1_* was also fine mapped and a underlying multi-gene complex was found to be the cause of effect of this QTL (Dixit et al. 2015). This study reports the introgression of *qDTY_3.2_* and *qDTY_12.1_* into Sabitri to develop drought-tolerant near isogenic lines (NILs) with high yield and preferred grain quality using marker-assisted backcross breeding (MABB).

## Materials and methods

This study was conducted at the Ziegler experiment station of the International Rice Research Institute (IRRI), Los Baños, Laguna, Philippines (14°13′N latitude, 121°15′E longitude, 21 m above mean sea level). The marker-assisted pyramiding of the two QTLs *qDTY_3.2_* and *qDTY_12.1_* in Sabitri and the development and testing of fixed lines were carried out from 2012 to 2016. Phenotypic screening of the NILs was conducted in the dry season (DS) of 2015 and 2016.

### Plant material

The QTLs *qDTY_3.2_* and *qDTY_12.1_* were detected in BC_1_F_3:5_ populations developed from the crosses IR 77298-5-6-18/2*Sabitri and IR 74371-46-1-1/2*Sabitri, respectively (Yadaw et al. 2013; Mishra et al. 2013). Sabitri is a popular long duration, high-yielding indica variety with good grain type but has a high susceptibility to drought. IR 77298-5-6-18 is a drought-tolerant NIL of IR64 and is a medium-duration, high-yielding indica line. IR 74371-46-1-1 is a medium-duration line developed from the cross Way Rarem/2*IR55419-04. This line was released as the drought-tolerant variety Sookha Dhan 1 for rainfed lowland ecosystem in Nepal in 2011 (Kumar et al. 2014). Lines from the two mapping populations showing the presence of the full segment of the QTLs were used as donors for the MAB program.

### Genotypic data and development of chromosome maps

Foreground selection was conducted using rice SSR markers and the final foreground screening of the selected NILs was conducted using SNP markers. A total of 3 and 6 markers closely linked to *qDTY_3.2_* and *qDTY_12.1_*, respectively, were used for foreground selection (Supplementary Table 1). The detailed protocol used to conduct the SSR genotyping is presented in Supplementary file 1. SNP genotyping was conducted using infinium 6 K SNP genotyping platform for the selected NILs to check their background recovery. The chromosome map of the NIL was constructed using GGT 2 (Van Berloo 2008).

### QTL introgression and selection

**Fig. 1:**
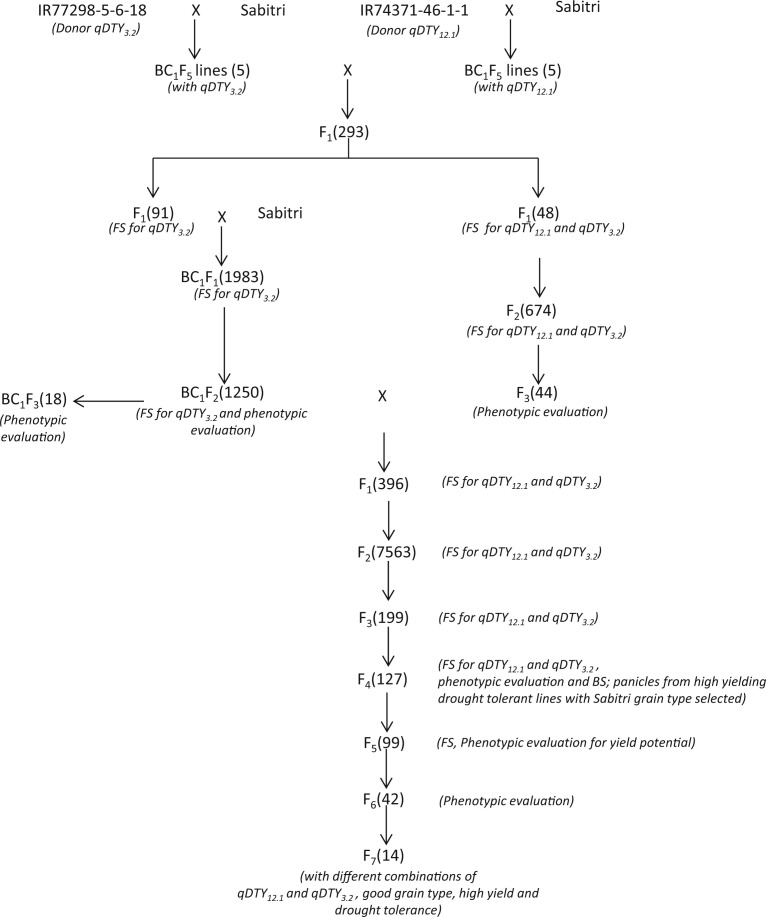
Marker-assisted Sabitri backcross breeding scheme used to develop drought-tolerant NILs of recipient parent Sabitri. BC (followed by the number as subscript) refers to the backcross generation; F (followed by the number as subscript) refers to the filial generation developed through selfing after the backcross. Numbers within parenthesis after each generation refers to the number of plants generated

**Fig. 2:**
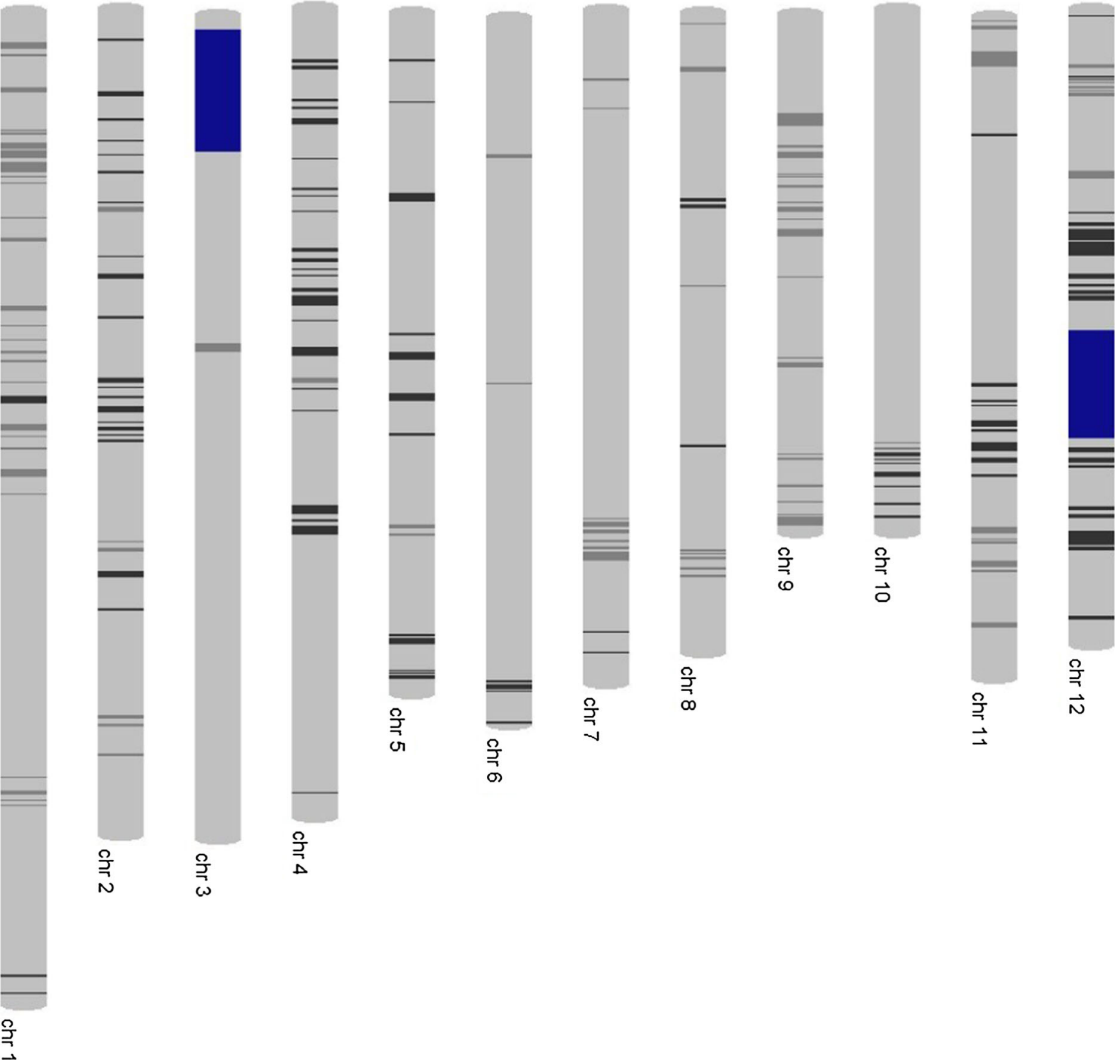
Graphical genotype of one of the NILs generated through 6 K infinium SNP genotyping. Light gray color shows recipient (Sabitri) allele, black color shows donor (IR74371-46-1-1 or IR77298-5-6-18) allele, and dark gray color shows heterozygotes. Regions colored in blue are the QTL regions. (Color figure online)

Figure 1 presents the crossing scheme used to pyramid *qDTY_3.2_* and *qDTY_12.1_* into Sabitri. BC_1_F_5_ lines with full segments of *qDTY_3.2_* and *qDTY_12.1_* from the mapping populations used to identify the QTLs were used as sources for the crossing program. Five lines from each population were crossed to develop a total of 293 F_1_ plants which were then genotyped with foreground markers for the two QTLs. Among the five sets of F_1_s developed, two sets were used for further crossing program: the first set (91 plants) segregated for *qDTY_3.2_* and showed good plant type and grain type and the second set (48 plants) segregated for both *qDTY_3.2_* and *qDTY_12.1_*, but the plant type differed from the Sabitri plant type. Plants were selected from these two sets based on the presence of QTL markers in heterozygote state and advanced in two different ways. The selected plants from the first set of F_1_s were backcrossed to Sabitri to develop a BC_1_F_1_ population segregating for *qDTY_3.2_*. The population was again subjected to foreground selection and the selected BC_1_F_1_s were used to develop a large BC_2_F_2_ population (1250 plants). From this population, plant selections were made based on the presence of *qDTY_3.2_*, plant type, and grain type. A total of 18 plants were advanced to the BC_1_F_3_ generation and multiplied. Plant selections segregating for both *qDTY_3.2_* and *qDTY_12.1_* from the second set of F_1_s were advanced to develop a large F_2_ population with 674 plants. These plants were genotyped with foreground markers for both QTLs and advanced to F_3_ generation. A total of 44 F_3_ lines were tested for yield potential and plant type and the selected high-yielding lines were advanced to F_4_. While these F_3_ lines contained the two QTLs, their plant type and grain type were not similar to that of Sabitri. The selected lines from this population were, thus, crossed to the plant selections from the BC_1_F_2_ population from the first set and a total of 396 F_1_ plants were developed and screened for the presence of both QTLs. F_1_ plants segregating for both QTLs were advanced to develop a large F_2_ population (7563 plants). This population was screened for the presence of both QTLs and the identified plants were then screened for similarity to Sabitri plant type and grain type resulting in 199 selected plants that were advanced to the F_3_ generation. The number of lines was further reduced to 127 based on their yield performance compared to Sabitri. Single plants selected from these 127 lines based on yield potential and grain type were used to develop a population of 127 F_4_ lines which were tested for their performance under drought and non-stress conditions. Six selected NILs from this experiment were also checked for background recovery. Graphical genotype of one of these NILs showing the introgressed QTLs and the background recovery is presented in Fig. 2. Apart from this, 99 panicle selections were made from the best high-yielding drought-tolerant lines based on their grain types for seed purity and the yield potential of these 99 lines was tested under the non-stress condition. A total of 42 F_6_ lines developed from the selected F_5_ lines were tested under non-stress and drought conditions. Finally, 14 drought-tolerant lines with high yield were selected and characterized for grain type for further screening in the target environment.

### Management of drought and non-stress experiments

The NILs selected from the backcross program were tested in a transplanted lowland ecosystem under reproductive-stage drought stress and non-stress conditions. Seedlings were raised in a wet-bed nursery. One 21-day-old seedling was transplanted per hill in the field-based rainout shelter facility at IRRI. For the stress trials, approximately 5 cm of standing water was maintained in the field up to 30 days after transplanting, after which the water was drained to initiate stress. For the non-stress trials, standing water was maintained up to 10 days before harvest. Field management was carried out as described by Venuprasad et al. (2009). In the drought stress experiments, severe stress was imposed in both years to ensure the selection of true drought-tolerant lines. In DS2015, the drought stress experiment was planted in an alpha lattice design in a plot size of 0.7 m^2^ with two replications. Under the non-stress condition, a single replication augmented randomized complete block design with a plot size of 1 m^2^ was used. Smaller plot sizes were maintained due to the limited amount of seeds harvested from plant selections in the previous season. However, continuous planting in single row plots allowed the precise measurement of the yield of the lines in both experiments. Such experimental layouts have been extensively used in drought stress experiments at IRRI with larger populations (Bernier et al. 2007; Venuprasad et al. 2009; Dixit et al. 2012b; Vikram et al. 2011). For further confirmation of the performance of these lines under drought and nonstress, selections from the best performing lines identified from the DS2015 experiments were screened in DS2016 using an alpha lattice design with two replications and plot sizes of 2.9 and 4.0 m^2^, respectively. Water table depth was monitored every day to keep track of the severity of drought in both years (Supplementary Fig. 1). Lifesaving irrigation was provided when the water table fell below 100 cm depth and 50% of the lines showed severe leaf rolling.

### Observations recorded

Days to 50% flowering (DTF), mean plant height at maturity (PH), and GY were recorded in all experiments. DTF was recorded as the number of days from seeding until the day when tillers of 50% of the plants in a plot started flowering. PH of three plants was measured at maturity by recording the length of the tallest tiller from ground level and averaging the values to get the mean PH. To get the GY, grains from each plot were harvested at physiological maturity, dried to a moisture content of about 14%, and weighed (Venuprasad et al. 2009). Yield per unit area was calculated using this weight for further analysis. Grain parameters were measured for 20 selected NILs and Sabitri from the non-stress harvest. Vernier caliper was used to measure grain length and width for 10 whole and de-hulled grains of the NILs and Sabitri and the length–width ratio was calculated. The kernel shape of the NILs and Sabitri was determined using the length–width ratio according to the standard evaluation system for rice (IRRI 2014). One hundred grains for each NIL and Sabitri were sampled randomly from the seed lot and weighed to determine the grain weight.

### Statistical analysis

CROPSTAT version 7.2.3 (http://archive.irri.org/science/software/cropstat.asp) was used to analyze the data of all experiments for the computation of means and standard error of difference (SED). Mixed model analysis was carried out using the model.

$$y_{ijk}=\mu +g_{i}+r_{j}+b_{lj}+e_{ijk}$$

where *μ* is the overall mean, *g_i_* is the effect of the ith genotype, *r_j_* is the effect of the jth replicate, *b_ij_* is the effect of the lth block within the jth replicate, and *e_ijk_* is the error. Effects for genotype were considered fixed and those for replicates and block were considered random for the analysis. Broad-sense heritability (*Η*) of the traits was calculated using the formula below:

H=σ2G′σ2G′+[σ2E(r)]

where $\sigma \frac{2}{G^{\prime }}$ is the genetic variance, $\sigma \frac{2}{E}$ is the plot residual variance, and r is the number of replications. Drought yield index′ (DYI) was calculated using the formula described by Raman et al. (2012) as follows:

(Yi)NS(Yi)S(G)NS(G)S

where *NS* and *S* refer to non-stress and stress, respectively, *Y_i_* is the mean yield of a genotype on the untransformed scale, and *G* is the geometric mean across genotypes. Phenotypic correlations of the three traits were calculated for the final set of lines screened in DS2016.

## Results

### MABB and NIL development

Figure 1 presents the marker-assisted selection procedure used to develop drought-tolerant NILs of Sabitri. The QTLs *qDTY_3.2_* and *qDTY_12.1_* were pyramided into Sabitri using BC_1_ lines from the two mapping populations in which these QTLs were identified. Marker-assisted selection was conducted at the start, however, once the QTLs were fixed, marker-assisted selection coupled with phenotypic evaluation were performed. Starting with F_2_ onwards, phenotypic characteristics such as plant type and grain type were taken into account during selection, along with the presence of QTLs. In the later stages, the lines were tested under drought and non-stress conditions to select high-yielding drought-tolerant NILs with similar grain quality to Sabitri. This approach proved suitable not only to introgress the major effect QTLs but also to phenotypically capture the effect of other favorable alleles and their combinations which may differ among the lines. This approach allowed the researchers to develop not only drought-tolerant lines with yield and grain quality similar to Sabitri but also other variants with higher yield potential and different grain type. Background screening with 6 K SNP platform for the best NIL IR106522-35-5-3 showed 92.1% genetic similarity to Sabitri.

### Phenotypic variation and trait correlations

Significant phenotypic variations for GY, DTF, and PH were observed in all trials (Table 1). Broad sense heritability for these traits ranged between 0.61 and 0.89 in DS2015 and between 0.81 and 0.96 in DS2016. The three parents showed similar yields under nonstress. However, under stress, IR 74371-46-1-1 was the highest yielding line followed by IR 77298-5-6-18 and then Sabitri in both seasons (Table 1). The reduction in yield was higher in DS2016 as compared to DS2015, indicating a higher severity of stress in this season. DTF of the three lines followed a similar sequence, with IR 74371-46-1-1 flowering earliest followed by IR 77298-5-6-18 and then Sabitri. PH patterns of the three parents differed under drought and non-stress conditions. Sabitri and IR 74371-46-1-1 showed similar PH (94 and 95 cm, respectively) while IR 77298-5-6-18 was relatively shorter (86 cm) under non-stress in DS2015. In DS2016, Sabitri (93 cm) and IR74371-46-1-1 (90 cm) showed taller plant type compared to IR77298-5-6-18 (89 cm). A high reduction in PH was observed for Sabitri under stress in both seasons, indicating its susceptibility towards drought. The reduction in PH for IR77298-5-6-18 was higher in DS2016 compared to DS2015 due to more severe stress levels. IR74371-46-1-1 showed the least height reduction in both seasons. The P (probability of difference between genotypes) values of all traits except nonstress GY in DS2015 were highly significant. The lesser degree of significance for GY under non-stress indicates a relatively lesser variation in yield under non-stress in this season despite the high variation for the other traits. However, under drought stress, the mean yield of progenies was 12 and 13 folds higher than that of Sabitri in DS2015 and 2016, respectively, showing the higher degree of tolerance of these lines. Apart from this, the percentage yield reduction of the progenies ranged between the two drought-tolerant donors. Significant differences for DTF and PH were observed between the parents and progenies under stress and non-stress conditions, hence, understanding their effect on yield becomes important. For this, phenotypic correlations among the three traits were calculated (Table 2). DTF and GY had a negative correlation under drought but a positive correlation under non-stress. This shows that the early flowering of the progenies gave them a yield advantage under drought while the late-duration lines showed an advantage under non-stress condition. Despite this, a large number of lines with earlier flowering showed higher yields compared to the recipient Sabitri under non-stress conditions. PH and GY had no significant correlation under non-stress conditions, however, their correlation was highly significant under stress, showing that yield advantage under stress was not related to PH per se but with the maintenance of PH and biomass under stress conditions.

**Table 1:** Analysis of variance for drought stress and non-stress experiments conducted in DS2015 and DS2016 showing means of parents and progeny

Season	Designation	Grain yield (kg ha ^1^) Non-stress Stress		Days to flowering Non-stress Stress		Plant height (cm) Non-Stress Stress		YR
DS2015	Sabitri	6747	48	103	113	94	48	99
	IR77298-5-6-18	6442	236	90	96	86	57	96
	IR74371-46-1-1	6790	1390	80	74	95	85	80
	Progeny	5711	564	86	88	81	61	90
	P	*	****	****	****	****	****	-
	SED	1069	271	3	4	4	5	-
	H	0.61	0.74	0.85	0.82	0.89	0.72	-
DS2016	Sabitri	4749	0	85	89	93	70	99
	IR77298-5-6-18	5220	113	79	87	89	64	99
	IR74371-46-1-1	3675	932	71	70	90	84	75
	Progeny	5209	290	77	79	90	70	94
	P	****	****	****	****	****	****	-
	SED	455	132	1	2	2	4	-
	H	0.83	0.81	0.96	0.92	0.95	0.90	-

YR percentage yield reduction compared to non-stress, P probability of difference between genotypes, SED standard error of difference, H heritability (broad sense)

*, **** Significant at 5%, and 0.01% P levels, respectively

**Table 2:** Phenotypic correlations between traits under drought stress and non-stress conditions in DS2016

	DTF (NS)	PH (NS)	GY (NS)	DTF (S)	PH (S)	GY (S)
DTF (NS)	1					
PH (NS)	0.39**	1				
GY (NS)	0.47**	0.07	1			
DTF (S)	0.65**	0.20	0.40**	1		
PH (S)	−0.14	0.70**	−0.32*	−0.22	1	
GY (S)	−0.43**	0.02	−0.06	−0.64**	0.42**	1

DTF days to 50% flowering, PH mean plant height at maturity (cm), GY grain yield (kg ha^–1^), S drought stress condition, NS nonstress condition

*, **** Significant at 5 and 1% levels, respectively

### Effect of QTL classes and performance of selected NILs

**Fig. 3:**
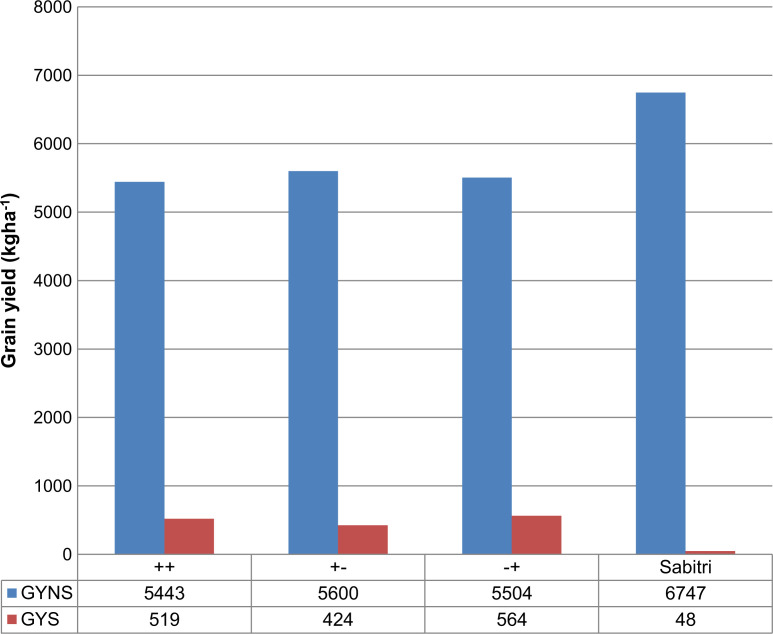
Mean grain yield for NILs possessing different combinations of *qDTY_3.2_* and *qDTY_12.1_* along with recipient parent Sabitri. ‘++’ class refers to lines with both QTLs, ‘+−’ refers to lines with *qDTY_3.2_* only, and ‘−+’ refers to lines with *qDTY_12.1_* only

The NILs screened in DS2015 were divided into three classes based on the presence of *qDTY_3.2_* and *qDTY_12.1_*: ‘++’ (with both QTLs), ‘+−’ (with *qDTY_3.2_* only), and ‘−+’ (with *qDTY_12.1_* only). To understand the effect of the QTL combinations, class mean yields were calculated. Similar yields were observed for all three classes under drought stress and non-stress conditions (Fig. 3). However, a slight advantage was observed for the ‘−+’ class compared to the other two classes in both conditions. Yield advantage over Sabitri was observed for all three classes under stress as majority of the lines yielded higher than Sabitri. However, under non-stress, the yield of the lines varied on both higher and lower sides of the yield of Sabitri. This led to a lower overall class mean yield of the different classes under non-stress as compared to Sabitri.

**Fig. 4:**
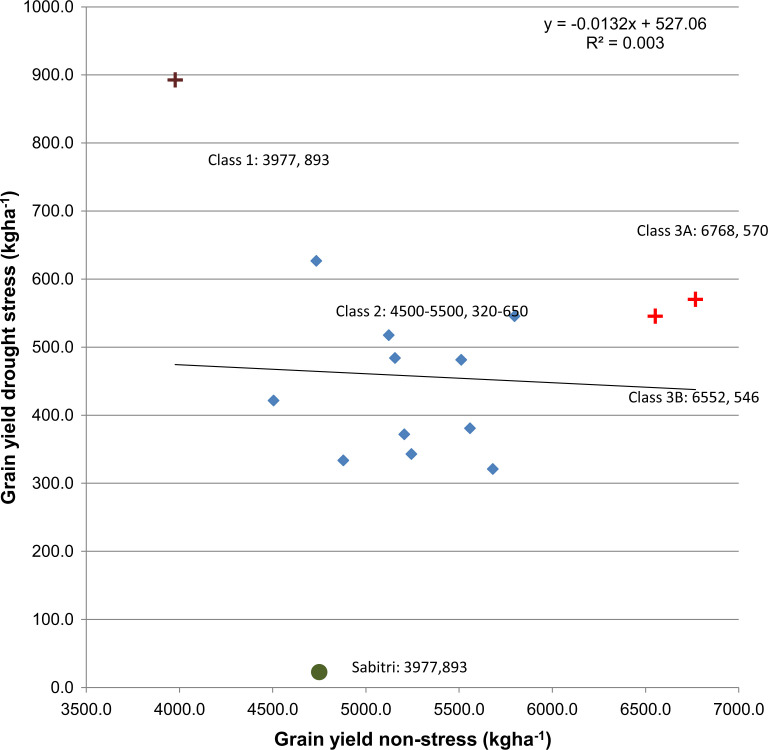
Scatter plot of yield under drought stress and non-stress conditions for 14 drought tolerant NILs presented in Table 3 along with Sabitri. ‘+’ indicates outliers with yield patterns different from the general trend while circle represents the yield pattern of Sabitri

A total of 14 NILs of Sabitri were identified based on the presence of the two QTLs and phenotypic performance (Table 3). Ten of these NILs were fixed for both QTLs, two were with a partial segment of *qDTY_12.1_*, one with a partial segment of *qDTY_3.2_*, and one with *qDTY_3.2_* only. These four NILs were selected due to their high yield under non-stress and tolerance to drought. While all these NILs out-yielded Sabitri many times higher under drought, different NIL classes were distinctly visible. These included NILs with high yield under drought stress (893 kg ha^–1^) but with low yield under non-stress (<3977 kg ha^–1^), those with moderate yield (320–650 kg ha^–^1) under stress and similar yield as Sabitri under non-stress (4500–5500 kg ha^–1^), and those with very high yield under non-stress (>6500 kg ha^–1^) but moderate yield (500–600 kg ha^–1^) under stress. The drought yield index (DYI) of Sabitri was much higher compared to any of the lines due to the large difference between its yields under non-stress and stress conditions. To further understand the relationship between the yields of these lines under non-stress and stress conditions, a regression analysis was conducted which showed no relation between the grain yields under both conditions (Fig. 4). However, outliers to this pattern were observed. IR106522-35-5-3-1-B and IR 106522-35-5-3-3-B showed high yield under both conditions and plotted far from the other lines on the scatter plot. Similarly, IR 106523-24-35-1-3-B both showed very high yield under drought but low yield under nonstress conditions and plotted separate from the other lines (Table 3; Fig. 4). It also had the earliest flowering time of all the lines which could account for the yield decline under non-stress. The coefficient of determination (R^2^) value for the regression model was very low (0.003), showing the independence of the yields in both conditions regardless of the presence of these outliers.

**Table 3:** Performance of selected NILs under drought stress and non-stress conditions in DS2016

Designation	Grain yield (kg ha^–1^)		YR	Days to flowering		FD	Plant height (cm)		PHD	DYI	QTL (*qDTY_3.2-qDTYj2.1_*)
	Non-stress	Stress		Non-stress	Stress		Non-stress	Stress			
IR 106522-37-5-3-1-B	5682	321	94	79	78	−1	88	58	30	1.1	++
IR 106526-39-21-1-1-B	4880	333	93	76	71	−5	87	65	22	0.9	++
IR 106529-20-40-3-1-B	5245	343	93	75	74	−1	85	68	17	0.9	+P
IR 106529-20-40-3-2-B	5206	372	93	78	77	−1	90	76	14	0.9	++
IR 106523-6-35-2-3-B	5559	381	93	75	79	4	85	73	12	0.9	++
IR 106523-23-8-1-2-B	4505	421	91	72	76	5	86	74	11	0.7	P+
IR 106522-35-45-2-1-B	5512	481	91	78	80	3	94	73	21	0.7	++
IR 106522-37-5-3-2-B	5156	484	91	74	78	4	80	59	22	0.6	+P
IR 106523-21-28-1-2-B	5123	517	90	77	72	−5	86	68	18	0.6	++
IR 106523-3-9-3-2-B	5798	545	91	75	75	0	82	63	18	0.6	++
IR 106522-35-5-3-1-B	6552	546	92	81	82	2	97	71	26	0.7	++
IR 106522-35-5-3-3-B	6768	570	92	80	81	1	93	77	15	0.7	++
IR 106522-41-8-3-B	4735	627	87	72	72	1	84	76	8	0.5	−+
IR 106523-24-35-1-3-B	3977	893	78	76	71	−6	109	102	6	0.3	++
Sabitri	4749	0	100	85	89	4	93	70	23	289.1	--
IR77298-5-6-18	5220	113	98	79	87	8	89	64	25	2.8	*qDTY_32_ donor*
IR74371-46-1-1	3675	932	75	71	70	−1	90	84	6	0.2	*qDTY_12.1 donor_*
Progeny	5209	290	94	77	79	2	90	70	20	1.1	
P	****	****	−	****	****	−	****	****	−	−	
SED	455	132	−	1	2	−	2	4	−	−	
H	0.83	0.81	−	0.96	0.92		0.95	0.9	−	−	

Stress grain yield of Sabitri was assumed 1 kg ha^–1^ to complete the calculation of DYI

DYI drought yield index, YR percentage yield reduction under stress compared to non-stress, FD difference in flowering time (days) under stress and non-stress, PHD difference in plant height (cm) under stress and non-stress conditions, P probability of difference between genotypes, SED standard error of difference, H heritability (broad sense)

*, **** Significant at 5 and 0.01% *P* levels respectively

The NILs were significantly early flowering compared to Sabitri under both conditions and had similar flowering time under non-stress in majority of the cases while some of them flowered earlier under stress. An exception to this were IR 106523-23-8-1-2-B, IR 106523-6-35-2-3-B, and IR 106522-37-5-3-2-B which showed a delay of 4–5 days in flowering under stress. Compared to the NILs, Sabitri showed a 5-day delay in flowering under stress. Plant height reductions were also observed for all NILs under the stress conditions (Table 3).

### Grain type of NILs

**Fig. 5:**
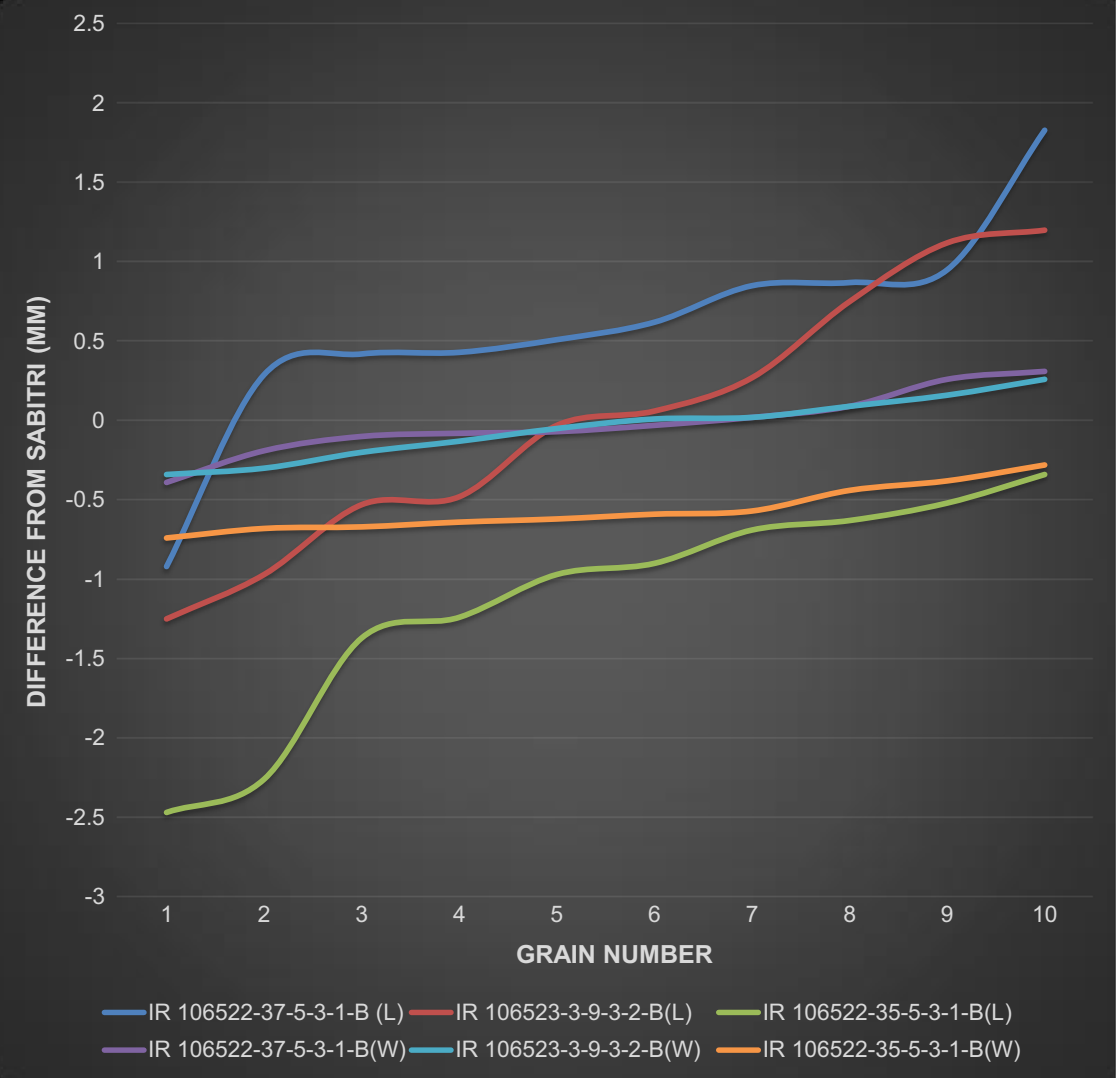
Grain length and width differences of three NILs from Sabitri for 10 grains. IR 106523-3-9-3-2-B showed the highest similarity with Sabitri, IR 106522-37-5-3-1-B had long and slender grains, while IR 106522-35-5-3-1-B had shorter and more slender grains compared to Sabitri

**Fig. 6:**
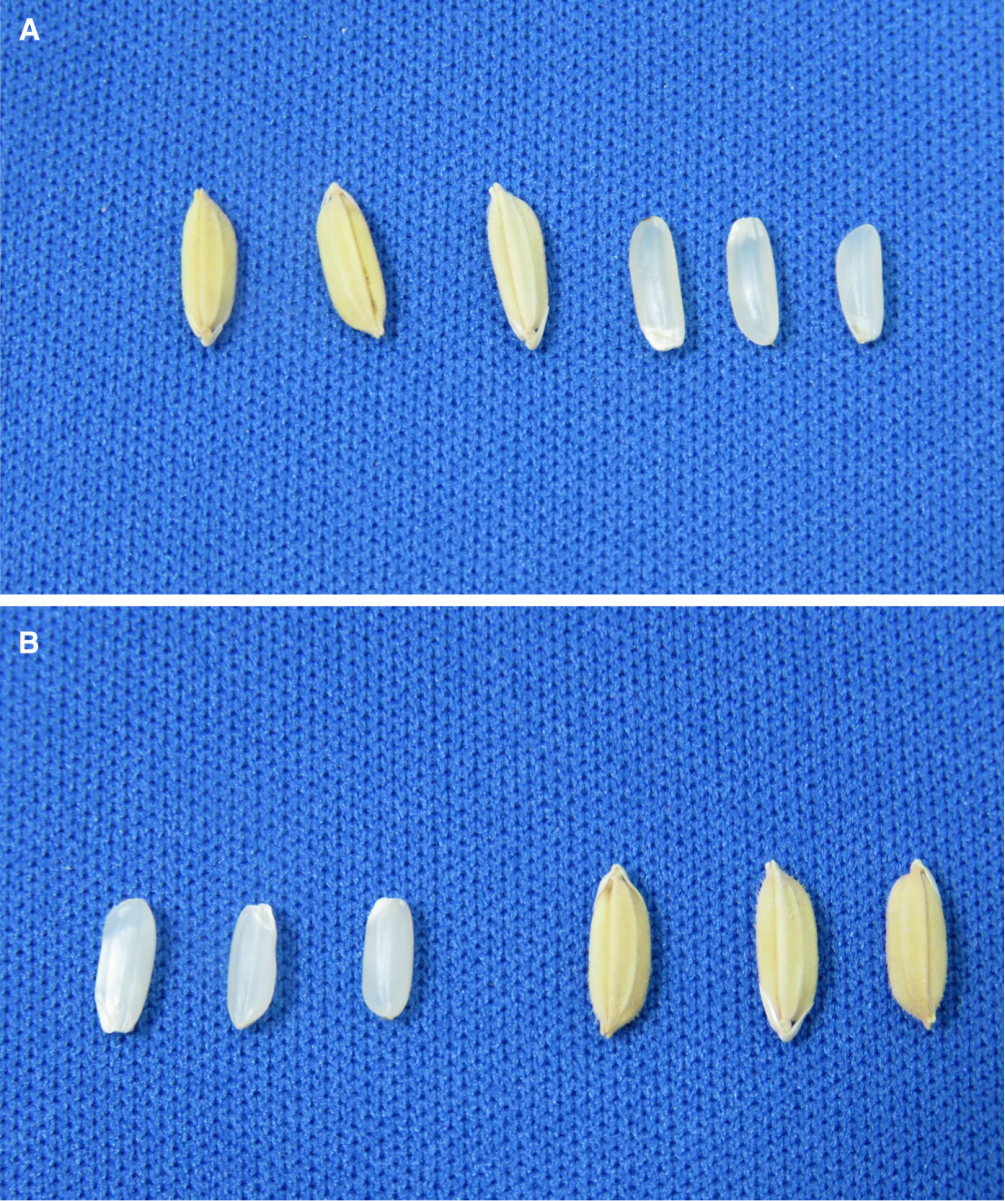
Grain type of A: Sabitri and B: IR106522-37-5-3 for whole and de-hulled grains

In general, the grain type of all the NILs was close to each other and majority of them were classified as medium or slender (Table 4). However, detailed grain measurements grouped the NILs into three different categories based on their grain shape difference compared to Sabitri. Figure 5 presents the difference of three NILs from Sabitri for grain length and width of 10 grains. IR 106523-3-9-3-2-B showed the lowest difference from Sabitri for both grain length and width. IR 106522-37-5-3-1-B had longer grains compared to Sabitri while their widths were similar. IR 106522-35-5-3-1-B, on the other hand, showed shorter grains with lesser grain width as compared to Sabitri. The grain shape of all the other NILs ranged between these two extremes. Further, the similarity of grain type and appearance of one of the NILs and Sabitri is presented in Fig. 6.

**Table 4:** Grain type variations observed in the NILs selected in DS2016

Line	Whole grain			De-hulled grain			100 grain weight (gm)	Grain shape
	Length (mm)	Width (mm)	L:W	Length (mm)	Width (mm)	L:W		
IR 106522-37-5-3-1-B	9.3	2.6	3.5	6.9	2.3	3.0	2.6	Slender
IR 106526-39-21-1-1-B	8.5	2.6	3.3	6.0	2.2	2.8	2.1	Slender
IR 106529-20-40-3-1-B	8.3	2.7	3.1	6.0	2.2	2.7	2.3	Slender
IR 106529-20-40-3-2-B	8.2	2.7	3.1	5.9	2.3	2.5	2.3	Slender
IR 106523-6-35-2-3-B	8.4	2.5	3.3	5.4	2.6	2.1	2.2	Slender
IR 106523-23-8-1-2-B	8.8	2.7	3.3	6.6	2.2	3.0	2.7	Slender
IR 106522-35-45-2-1-B	8.0	2.7	3.0	5.7	2.3	2.5	2.2	Slender
IR 106522-37-5-3-2-B	8.7	2.7	3.3	5.9	2.2	2.6	2.4	Slender
IR 106523-21-28-1-2-B	8.6	2.7	3.2	6.1	2.3	2.6	2.3	Slender
IR 106523-3-9-3-2-B	8.7	2.6	3.3	6.1	2.2	2.8	2.4	Slender
IR 106522-35-5-3-1-B	7.6	2.1	3.6	5.8	2.4	2.5	2.3	Slender
IR 106522-35-5-3-3-B	8.1	2.7	3.0	5.7	2.4	2.4	2.3	Slender
IR 106522-41-8-3-B	8.2	2.7	3.1	6.3	2.2	2.9	2.6	Slender
IR 106523-24-35-1-3-B	9.1	2.7	3.3	6.3	2.3	2.8	2.5	Slender
Sabitri	8.7	2.7	3.3	6.2	2.3	2.7	2.3	Slender

L:W length–width ratio

## Discussion

MABB has been used extensively in rice breeding as an approach to incorporate genes/QTLs of interest in popular rice cultivars. Several reports of marker-assisted introgression of genes/QTLs related to grain type and quality, biotic and abiotic stress tolerance, and yield-related traits are available, which include the development of disease-resistant rice cultivars (Huang et al. 1997; Hittalmani et al. 2000; Singh et al. 2001), high-yielding NILs of rice cultivars (Zhang et al. 2006), and high-quality rice lines (Yi et al. 2009; Joseph et al. 2004). For abiotic stress tolerance, the most famous example is the introgression of Sub1 into popular rice cultivars (Septiningsih et al. 2009; Neeraja et al. 2007). Similarly, the Saltol QTL for salinity tolerance has been transferred into elite rice varieties to develop their salinity-tolerant version (Linh et al. 2012). For drought, the introgression of QTLs controlling nodal root diameter has been reported (Steele et al. 2006). However, despite a number of MABB programs previously conducted for drought tolerance in rice, only a few reported large and consistent effects. This has mainly been attributed to the complex genetic control of drought tolerance. In this study, NILs of Sabitri that possessed a combination of *qDTY_3.2_* and *qDTY_12.1_* were developed and tested under non-stress and drought stress conditions. The donor parent IR 74371-46-1-1 (for *qDTY_12.1_*) showed higher yield compared to most of the introgressed lines with *qDTY_12.1_* in DS2015 and DS2016. This indicates the presence of additional QTLs in IR 74371-46-1-1 or the existence of a positive interaction between the QTLs and their genetic background that was not captured when the *qDTY_12.1_* was introgressed to the Sabitri background. The NIL IR 106523-24-35-1-3-B seemed to have captured most of the positive alleles from the donor parents as its yield was very similar to that of IR 74371-46-1-1 under drought and higher under non-stress. On the other hand, most of the progenies showed higher yields compared to the donor parent IR 77298-5-6-18 under drought, demonstrating the enhancement of positive alleles in the progeny lines. QTL class analysis showed similar yields of the lines with a full segment of the two QTLs (Fig. 3). However, a slightly higher yield was recorded for those with only *qDTY_12.1_* under both conditions.

The decline in yield under non-stress may be attributed to the presence of *qDTY_3.2_* in these two classes. *qDTY_3.2_* is tightly linked to the HD9 gene (Lin et al. 2002) which has a large effect on DTF. Its effect on flowering time leads to higher tolerance under drought but low yield under non-stress. However, in cases where the linkage between *qDTY_3.2_* and flowering time is broken, a high yield under both conditions is achieved. The effect of the linkage between *qDTY_3.2_* and DTF on yield potential is also clear by looking at the selected NILs (Table 3; Fig. 4). The NIL IR 106523-24-35-1-3-B which had both QTLs showed early flowering and highest yield under drought. However, its yield under non-stress was lower than that of the other NILs. On the other hand, several other NILs with the same QTL combinations were found to have relatively longer flowering time and low or no yield decline under non-stress. However, their advantage under drought was relatively lesser, showing the advantage of early flowering under drought as well as the effect of successful elimination of linkage drags on the performance of the line.

Knowing the source of the tolerant allele for *qDTY_3.2_* is also important to determine the level of yield decline. In cases where very early flowering landraces were the sources of this QTL, a higher decline in yield was observed under non-stress (Dixit et al. 2012b). However, in this study, the donor line (IR 77298-5-6-18) used is a high-yielding medium-duration NIL of IR64, hence, the negative effect was relatively small. A similar observation was previously recorded with this QTL in the original mapping study (Yadaw et al. 2013), which clearly indicated that days to maturity of the donor line contributing the *qDTY_3.2_* QTL is an important factor in determining the yield potential of introgressed lines under non-stress condition. Breeders should, therefore, select a donor line with *qDTY_3.2_* with medium maturity to avoid yield penalty under non-stress condition.

Other QTLs such as qDTY_3.1_ and qDTY_9.1_ have also been reported to be associated with yield decline under non-stress (Venuprasad et al. 2009; Dixit et al. 2012a, b). Similarly, other QTLs like *qDTY_1.1_* have been reported to show linkage with other undesirable traits like tall plant height (Vikram et al. 2015). The reduction of some of the progenies under non-stress in the present study indicates the existence of similar undesirable linkages for *qDTY_3.2_*. Until these DTY QTLs are fine mapped, undesirable linkages are identified and broken, and a gene or a set of genes underlying these QTLs are identified with linked markers, MABB programs that aim to improve yield under drought should use a combination of marker-assisted and phenotypic selections for yield potential to avoid undesirable linkage drags.

Sufficiently large populations must be developed and repeated cycles of selections must be conducted to develop and identify recombinants with favorable alleles that could produce yield advantages under both drought and non-stress conditions. This study not only identified drought-tolerant NILs of Sabitri but also combined the high yield potential of Sabitri under nonstress with drought tolerance. It involved the development of large populations segregating for the two QTLs which also allowed the segregation of other important traits such as grain type and yield potential. We combined marker-assisted selection with phenotypic selection to develop high-yielding NILs with drought tolerance and good grain quality. While majority of the NILs identified in the study showed early flowering and reduced plant height compared to Sabitri, the presence of variants for grain type and yield potential was also observed (Tables 3, 4). This clearly demonstrates the possibility of combining these traits together through systematic breeding programs with sufficiently large populations. In this study, drought-tolerant lines with higher yield compared to Sabitri under non-stress conditions with earliness of up to 11 days and similar plant type were developed. Other sets of variants in the NIL population included lines with low yield under non-stress but with high yield under drought. As both sets of lines showed similar DTF and possessed a high percentage of the Sabitri background, these could be ideal parent materials in developing mapping populations to determine yield limiting factors. Similarly, variants with the Sabitri grain type, longer than Sabitri type grains, and shorter grain type were identified (Fig. 5). Throughout the study, the focus was on the selection for QTLs and on the phenotypic performance of the lines as well as their grain type while background genotyping was conducted at later stages of the MABB program. This allowed the identification not only of lines similar to Sabitri but also of the variants discussed above.

The NILs developed can be used as a replacement for Sabitri in rainfed areas of Nepal and the presence of different variants allows a more widespread utilization of these lines. For example, lines with high yield under drought, moderate non-stress yield, and good grain quality can be used as parent material for future breeding programs as a replacement for landraces which lead to high linkage drag. These lines can also be deployed to upland areas where low-yielding landraces dominate cultivation due to high risk of drought. The higher yield potential of these lines compared to the landraces, superior grain quality, and high tolerance to drought may provide an additional advantage to farmers in such areas. Similarly, these NILs with very high non-stress yield and moderate yield under drought may be deployed to rainfed lowland or irrigated areas where mild intensities of drought are common. These lines can, thus, provide rice yield stability in Nepal and give farmers more flexibility to grow good quality rice without yield losses due to drought.

## Conclusion

High-yielding drought-tolerant NILs of Sabitri were developed and screened in this study. Two QTLs (*qDTY_3.2_* and *qDTY_12.1_*) previously reported in the background of this variety were introgressed and pyramided to develop the NILs. Marker-assisted backcross breeding coupled with phenotypic selection for yield potential and grain type allowed the development of early maturing NILs of Sabitri with similar yield potential and much higher tolerance to drought. Several different variants of NILs in terms of yield potential, drought tolerance, and grain quality were developed. These lines could be tested and deployed according to their suitability and the severity of drought in Nepal and other countries in South Asia not only as a replacement for Sabitri but also as replacement for other varieties with high susceptibility to drought.

## Notes

**Open Access** This article is distributed under the terms of the Creative Commons Attribution 4.0 International License (http://creativecommons.org/licenses/by/4.0/), which permits unrestricted use, distribution, and reproduction in any medium, provided you give appropriate credit to the original author(s) and the source, provide a link to the Creative Commons license, and indicate if changes were made.

## References

[cit0001] Bernier J, Kumar A, Venuprasad R, Spaner D, Atlin GN (2007) A large-effect QTL for grain yield under reproductive-stage drought stress in upland rice. Crop Sci 47:507–516

[cit0002] Dixit S, Swamy BPM, Vikram P, Ahmed HU, Sta Cruz MT, Amante M, Atri D, Leung H, Kumar A (2012a) Fine mapping of QTLs for rice grain yield under drought reveals sub-QTLs conferring a response to variable drought severities. Theor Appl Genet 125:155–169 22361948 10.1007/s00122-012-1823-9

[cit0003] Dixit S, Swamy BPM, Vikram P, Bernier J, Sta Cruz MT, Amante M, Atri D, Kumar A (2012b) Increased drought tolerance and wider adaptability of *qDTY_12.1_* conferred by its interaction with qDTY_2.3_and *qDTY_3.2_*. Mol Breed 30:1767–1779

[cit0004] Dixit S, Singh A, Kumar A (2014a) Rice breeding for high grain yield under drought: a strategic solution to a complex problem. Int J Agronomy. doi:10.1155/2014/863683

[cit0005] Dixit S, Singh A, Sta Cruz MT, Maturan PT, Amante M, Kumar A (2014b) Multiple major QTL lead to stable yield performance of rice cultivars across varying drought intensities. BMC Genet 15:16 24491154 10.1186/1471-2156-15-16 PMC3933981

[cit0006] Dixit S, Huang BE, Sta Cruz MT, Maturan PT, Ontoy JCE, Kumar A (2014c) QTLs for tolerance of drought and breeding for tolerance of abiotic and biotic stress: an integrated approach. PLoS ONE 9(10):e109574 25314587 10.1371/journal.pone.0109574 PMC4196913

[cit0007] Dixit S, Biswal AK, Min A, Henry A, Oane RH, Raorane ML, Longkumer T, Pabuayon IM, Mutte SK, Vardarajan AR, Miro B, Govindan G, Albano-Enriquez B, Pueffeld M, Sreenivasulu N, Slamet-Loedin I, Sundarvelpandian K, Tsai Y-C, Raghuvanshi S, Hsing YC, Kumar A, Kohli A (2015) Action of multiple intra-QTL genes concerted around a co-localized transcription factor underpins a large effect QTL. Nat Sci Rep 5:15183 10.1038/srep15183 PMC4623671 26507552

[cit0008] Ghimire KH, Quiatchon LA, Vikram P, Swamy BPM, Dixit S, Ahmed H, Hernandez JE, Borromeo TH, Kumar A (2012) Identification and mapping of a QTL (*qDTY_1.1_*) with a consistent effect on grain yield under drought. Field Crops Res 131:88–96

[cit0009] Hittalmani S, Parco A, Mew TV, Zeigler RS, Huang N (2000) Fine mapping and DNA marker-assisted pyramiding of the three major genes for blast resistance in rice. Theor App Genet 100:1121–1128

[cit0010] Huang N, Angeles ER, Domingo J, Magpantay G, Singh S, Zhang G, Kumaravadivel N, Bennett J, Khush GS (1997) Pyramiding of bacterial blight resistance genes in rice: marker-assisted selection using RFLP and PCR. Theor App Gen 95:313–320

[cit0011] IRRI (International Rice Research Institute) (2014) Standard Evaluation System for rice (SES), 5th edn. International Rice Research Institute, Los Baños

[cit0012] Joseph M, Gopalakrishnan S, Sharma RK, Singh VP, Singh AK, Singh NK, Mohapatra T (2004) Combining bacterial blight resistance and Basmati quality characteristics by phenotypic and molecular marker-assisted selection in rice. Mol Breed 13:377–387

[cit0013] Kumar V, Ladha JK (2011) Direct seeding of rice: recent developments and future research needs. Adv Agron 111:13. doi:10.1016/B978-0-12-387689-8.00001-1

[cit0014] Kumar A, Verulkar SB, Dixit S, Chauhan B, Bernier J, Venuprasad R, Zhao D, Shrivastava MN (2009) Yield and yield-attributing traits of rice (*Oryza sativa* L.) under lowland drought and suitability of early vigor as a selection criterion. Field Crops Res 114:99–107

[cit0015] Kumar A, Dixit S, Henry A (2013) Marker-assisted introgression of major QTLs for grain yield under drought in rice. In: Varshney RK, Tuberosa R (eds) Translational genomics for crop breeding: abiotic stress, yield, and quality, 1st ed. pp 47–64

[cit0016] Kumar A, Dixit S, Ram T, Yadaw RB, Mishra KK, Mandal NP (2014) Breeding high-yielding drought-tolerant rice: genetic variations and conventional and molecular approaches. J Exp Bot 65(21):6265–6278 25205576 10.1093/jxb/eru363 PMC4223988

[cit0017] Lin H, Ashihikari M, Yamanouchi U, Sasaki T, Yano M (2002) Identification and characterization of a quantitative trait locus, HD9, controlling heading date in rice. Breed Sci 52:35–41

[cit0018] Linh LH, Linh TH, Xuan TD, Ham LH, Ismail AM, Khanh TD (2012) Molecular Breeding to Improve Salt Tolerance of Rice (*Oryza sativa* L.) in the Red River Delta of Vietnam. Int J Plant Gen. doi:10.1155/2012/949038 PMC3544250 23326259

[cit0019] Mishra KK, Vikram P, Yadaw RB, Swamy BPM, Dixit S, Sta Cruz MT, Maturan P, Marker S, Kumar A (2013) qDTY 12.1: a locus with a consistent effect on grain yield under drought in rice. BMC Genet 14:12 23442150 10.1186/1471-2156-14-12 PMC3616849

[cit0020] Molden D, Frenken K, Barker R, de Fraiture C, Mati B, Svendsen M, Sadoff C, Finlayson CM, Attapatu S, Giordano M, Inocencio A, Lannerstad M, Manning N, Molle F, Smedema B, Vallée D (2007) Trends in water and agriculture development. Water for food, water for life: a comprehensive assessment of water management in agriculture. International Water Management Institute, Colombo, and London: Earthscan, pp. 57–89

[cit0021] Neeraja CN, Maghirang-Rodriguez R, Pamplona A, Heuer S, Collard BCY, Septiningsih EM, Vergara G, Sanchez D, Xu K, Ismail AM, Mackill DJ (2007) A marker-assisted backcross approach for developing submergence-tolerant rice cultivars. Theor Appl Genet 115:767–776 17657470 10.1007/s00122-007-0607-0

[cit0022] Palanog AD, Swamy BPM, Shamsudin NAA, Dixit S, Hernandez JE, Boromeo TH, Sta Cruz PC, Kumar A (2014) Grain yield QTLs with consistent-effect under reproductive-stage drought stress in rice. Field Crops Res 161:46–54

[cit0023] Raman A, Verulkar SB, Mandal NP, Variar M, Shukla VD, Dwivedi JL, Singh BN, Singh ON, Swain P, Mall AK, Robin S, Chandrababu R, Jain A, Ram T, Hittalmani S, Haefele S, Piepho H-P, Kumar A (2012) Drought yield index to select high yielding rice lines under different drought stress severities. Rice 5:31 27234249 10.1186/1939-8433-5-31 PMC5520844

[cit0024] Sandhu N, Singh A, Dixit S, Sta Cruz MT, Maturan PC, Jain RK, Kumar A (2014) Identification and mapping of stable QTL with main and epistasis effect on rice grain yield under upland drought stress. BMC Genet 15:63 24885990 10.1186/1471-2156-15-63 PMC4048250

[cit0025] Septiningsih EM, Pamplona AM, Sanchez DL, Neeraja CN, Vergara GV, Heuer S, Ismail AM, Mackill DJ (2009) Development of submergence-tolerant rice cultivars: the Sub1 locus and beyond. Ann Bot 103:151–160 18974101 10.1093/aob/mcn206 PMC2707316

[cit0026] Singh S, Sidhu JS, Huang N, Vikal Y, Li Z, Brar DS, Dhaliwal HS, Khush GS (2001) Pyramiding three bacterial blight resistance genes (*xa5, xa13* and *Xa21*) using marker-assisted selection into *indica* rice cultivar PR106. Theor App Genet 102:1011–1015

[cit0027] Steele KA, Price AH, Shashidhar HE, Witcombe JR (2006) Marker-assisted selection to introgress rice QTLs controlling root traits into an Indian upland rice variety. Theor App Genet 112:208–221 10.1007/s00122-005-0110-4 16208503

[cit0028] Swamy BPM, Ahmed HU, Henry A, Mauleon R, Dixit S, Vikram P, Tilatto R, Verulkar SB, Perraju P, Mandal NP, Variar M, Robin S, Chandrababu R, Singh ON, Dwivedi JL, Das SP, Mishra KK, Yadaw RB, Aditya TL, Karmakar B, Satoh K, Moumeni A, Kikuchi S, Leung H, Kumar A (2013) Genetic, physiological, and gene expression analyses reveal that multiple QTL enhance yield of rice mega-variety IR64 under drought. PLoS ONE 8(5):e62795 23667521 10.1371/journal.pone.0062795 PMC3648568

[cit0029] Van Berloo R (2008) GGT 2.0: versatile software for visualization and analysis of genetic data. J Heredity 99(2):232–236 10.1093/jhered/esm109 18222930

[cit0030] Venuprasad R, Lafitte HR, Atlin GN (2007) Response to direct selection for grain yield under drought stress in rice. Crop Sci 47:285–293

[cit0031] Venuprasad R, Sta Cruz MT, Amante M, Magbanua R, Kumar A, Atlin GN (2008) Response to two cycles of divergent selection for grain yield under drought stress in four rice breeding populations. Field Crops Res 107:232–244

[cit0032] Venuprasad R, Dalid CO, Del Valle M, Zhao D, Espiritu M, Sta Cruz MT, Amante M, Kumar A, Atlin GN (2009) Identification and characterization of large-effect quantitative trait loci for grain yield under lowland drought stress in rice using bulk-segregant analysis. Theor Appl Genet 120:177–190 19841886 10.1007/s00122-009-1168-1

[cit0033] Vikram P, Swamy BPM, Dixit S, Ahmed HU, Sta Cruz MT, Singh AK, Kumar A (2011) *qDTY_1.1_*, a major QTL for rice grain yield under reproductive-stage drought stress with a consistent effect in multiple elite genetic backgrounds. BMC Genet 12:89 22008150 10.1186/1471-2156-12-89 PMC3234187

[cit0034] Vikram P, Swamy BPM, Dixit S, Singh R, Singh BP, Miro B, Kohli A, Henry A, Singh NK, Kumar A (2015) Drought susceptibility of modern rice varieties: an effect of linkage of drought tolerance with undesirable traits. Sci Rep 5:14799. doi:10.1038/srep14799 26458744 PMC4602206

[cit0035] Yadaw RB, Dixit S, Raman A, Mishra KK, Vikram P, Swamy BPM, Sta Cruz MT, Maturan PT, Pandey M, Kumar A (2013) A QTL for high grain yield under lowland drought in the background of popular rice variety Sabitri from Nepal. Field Crops Res 144:281–287

[cit0036] Yi M, New KT, Vanavichit A, Chai-arree W, Toojinda T (2009) Marker assisted backcross breeding to improve cooking quality traits in Myanmar rice cultivar Manawthukha. Field Crops Res 113:178–186

[cit0037] Zhang Y, Luo L, Xu C, Zhang Q, Xing Y (2006) Quantitative trait loci for panicle size, heading date and plant height co-segregating in trait-performance derived near-isogenic lines of rice (*Oryza sativa*). Theor Appl Genet 113:361–368 16791702 10.1007/s00122-006-0305-3

