# Supplementary material for: Marker-assisted breeding to develop the drought-tolerant version of Sabitri, a popular variety from Nepal
Source: Euphytica. 2017 Jul 24;213:184. doi: 10.1007/s10681-017-1976-3 (PMC7734194; doi:10.1007/s10681-017-1976-3)
Supplement: Supplementary file 1 [file EUP-213-184-s001.pdf]

SSR genotyping protocol used for foreground selection during the NIL development process

For SSR genotyping, DNA was extracted from leaf samples collected from 2-week-old plants. Young leaf samples were collected and freeze-dried. Geno-grinder was used to grind these leaf samples and DNA was extracted using a modified CTAB method **(Murray and Thomson, 1980)** in deep-well plates. 0.8% agarose gel was used to check the quality and quantity of the extracted DNA and samples were then diluted to a final concentration of 20 ng μL^{-1} with TE (Tris-EDTA) buffer. The method described by **Panaud et al. (1996)** was used to perform polymerase chain reaction (PCR). Upon completion of PCR, 4 µL of 6X loading dye was added to the product and 4 µL of this solution mix was loaded into an 8% (w/v) polyacrylamide gel **(Sambrook et al., 1989)** for size separation of the amplified DNA fragments. The mini vertical electrophoresis system (CBS Scientific, model MGV-202–33) was used to conduct the electrophoresis. SYBR® Safe gel stain (Invitrogen) was used to stain the separated DNA fragments. The fragments were then visualized using a UV trans-illuminator.

Sambrook, J., Fritsch, E.F., Maniatis, T., 1989. Molecular cloning: a laboratory manual 2nd edn. Cold Spring Harbor, New York, USA.

Panaud, O., Chen, X., McCouch, S. 1996. Development of microsatellite markers and characterization of simple sequence length polymorphism (SSLP) in rice (*Oryza sativa* L.). Mol. Gen. Genet, 252:597–607.

Murray, M.G., Thompson, W.F., 1980. Rapid isolation of high molecular weight plant DNA. Nucleic Acids Res. 8, 4321–4326.
