# Supplementary material for: Marker-assisted breeding to develop the drought-tolerant version of Sabitri, a popular variety from Nepal
Source: Euphytica. 2017 Jul 24;213:184. doi: 10.1007/s10681-017-1976-3 (PMC7734194; doi:10.1007/s10681-017-1976-3)
Supplement: Supplementary file 2 [file EUP-213-184-s002.docx]

**Supplementary Table 1:** Rice microsatellite markers linked to *qDTY_3.2_* and *qDTY_12.1_*.

| **QTL** | **Marker** | **Forward primer** | **Reverse primer** | **PCR product size (bp)** |
| --- | --- | --- | --- | --- |
| *qDTY_3.2_* | RM569 | GACATTCTCGCTTGCTCCTC | TGTCCCCTCTAAAACCCTCC | 175 |
|  | RM231 | CCAGATTATTTCCTGAGGTC | CACTTGCATAGTTCTGCATTG | 182 |
|  | RM517 | GGCTTACTGGCTTCGATTTG | CGTCTCCTTTGGTTAGTGCC | 266 |
| *qDTY_12.1_* | RM28048 | TTCAGCCGATCCATTCAATTCC | GCTATTGGCCGGAAAGTAGTTAGC | 93 |
|  | RM28099 | TGTGCGGATGCGGGTAAGTCC | CCACCTGTCAACCACCGAAACC | 120 |
|  | RM28130 | CAGCAGACGTTCCGGTTCTACTCG | AGGACGGTGGTGGTGATCTGG | 175 |
|  | RM511 | CTTCGATCCGGTGACGAC | AACGAAAGCGAAGCTGTCTC | 130 |
|  | RM28166 | TGCTTGCAAACATTGCTTCTGG | ACTGATGTACTGAACACGGGAAGG | 195 |
|  | RM28199 | CGGCTTAGGGAGCGTCTGTAGG | GCATGCTAGTATGGCCACCATATTCC | 179 |
