# Supplementary figures and images for: Marker-assisted breeding to develop the drought-tolerant version of Sabitri, a popular variety from Nepal
Source: Euphytica. 2017 Jul 24;213:184. doi: 10.1007/s10681-017-1976-3 (PMC7734194; doi:10.1007/s10681-017-1976-3)

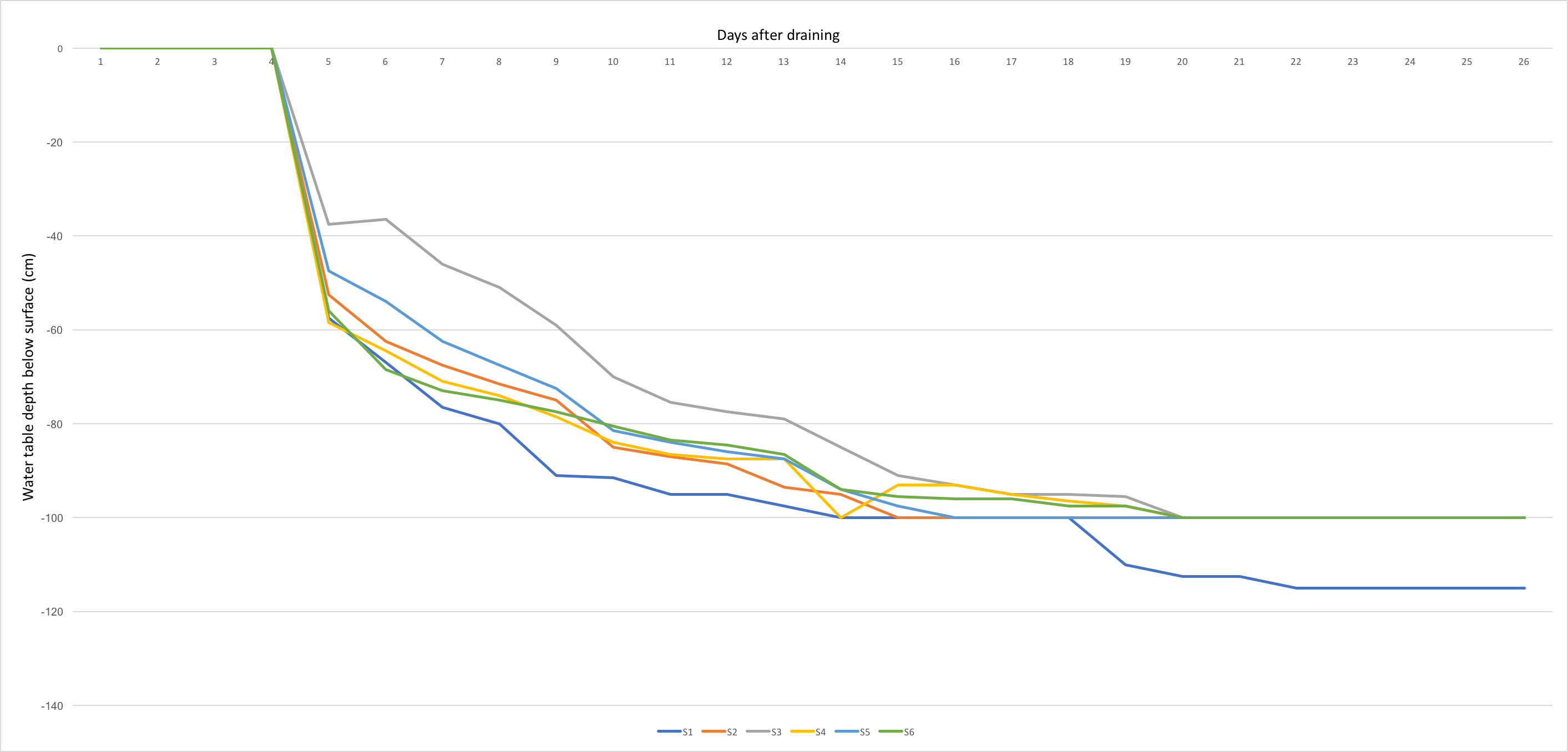

Supplement: Supplementary file 3 [file EUP-213-184-s003.png]
